# An External Focus of Attention Enhances Manual Tracking of Occluded and Visible Targets

**DOI:** 10.3389/fpsyg.2012.00591

**Published:** 2013-01-18

**Authors:** Matthew Schlesinger, Jared Porter, Robert Russell

**Affiliations:** ^1^Southern Illinois University CarbondaleCarbondale, IL, USA

**Keywords:** focus of attention, visuo-motor control, manual tracking, spatial working memory, occluded motion perception

## Abstract

The present study investigated the enhancement effects of an external focus of attention (FOA) in the context of a manual tracking task, in which participants tracked both visible and occluded targets. Three conditions were compared, which manipulated the distance of the FOA from the participant as well as the external/internal dimension. As expected, an external FOA resulted in lower tracking errors than an internal FOA. In addition, analyses of participants’ movement patterns revealed a systematic shift toward higher-frequency movements in the external FOA condition, consistent with the idea that an external FOA exploits the natural movement dynamics available during skilled action. Finally, target visibility did not influence the effect of focused attention on tracking performance, which provides evidence for the proposal that the mechanisms that underlie FOA do not depend directly on vision.

## Introduction

Practitioners (e.g., teachers, therapists, coaches, etc.) constantly look for techniques they can easily adopt to improve the performance of the individuals they are working with (e.g., students, patients, athletes, etc.). Numerous studies have been conducted in the last 15 years investigating how verbal instructions are used to influence how a person focuses their attention while executing motor skills (for a review, see Wulf, 2012). The common method used in this line of research has compared motor performance following verbal instructions or feedback designed to promote the participant to focus their attention internally, or externally. When a participant utilizes an internal focus of attention (FOA) they are consciously focusing on the movement characteristics of their body. However, when an external FOA is adopted the mover directs conscious attention toward the result of the movement on the environment (Wulf et al., 1998). For example, if a teacher was instructing a child how to correctly write letters in the alphabet (e.g., A, B, C, etc.), the teacher might instruct the student to be mindful of how to correctly move their hand and wrist to achieve the correct stroke to write the letter. This form of instruction would elicit an internal FOA because it prompts the child to attend to the movements of the hand and wrist. In contrast, the teacher could also instruct the child to focus his or her attention on the tip of the pencil and the stroke it is making on the paper. This form of instruction encourages an external FOA because is references the result of the movement rather than the movement of the hand.

Several lines of converging evidence have been reported supporting the use of instructions and feedback to encourage the adoption of an external FOA in a variety of practice and learning contexts (Wulf, 2012). These benefits have been investigated using a variety of object manipulation tasks such as playing a musical instrument (Duke et al., 2011), hitting a golf ball to a target (Wulf and Su, 2007), and shooting a basketball (Zachry et al., 2005). The enhancement has also been observed in complex whole body movements requiring multi-limb coordination such as vertical jump and reach (Wulf et al., 2010), horizontal jumping (Porter et al., 2010b, 2012; Wu et al., 2012), plyometrics training (Makaruk et al., 2012), and agility performance (Porter et al., 2010a). Additionally, the benefits of using an external FOA have been reported in non-typical populations such as patients with Parkinson disease (Wulf et al., 2009), cancer patients suffering from “chemo-brain” (Porter and Anton, 2011), and children with intellectual disabilities (Chiviacowsky et al., 2012).

In addition to examining the effects of an internal versus an external FOA, researchers have also investigated the “distance” effect of adopting an external FOA. The question of interest in this line of research is: if an external FOA benefits motor performance, then are performance benefits magnified when attention is directed externally at greater distances from the body? This question was initially investigated by McNevin et al. (2003). In that study participants performing a dynamic balance task were asked to focus their attention internally toward keeping their feet horizontal or externally on markers placed directly in front of their feet (i.e., external-near). These conditions were compared to separate external conditions that instructed participants to focus externally at greater distances toward markers that were located 26 cm away from their feet (i.e., external-far). Results indicated that balance performance improved as participants directed their attention externally at greater distances from their body. In a recent study, Porter et al. (2012) tested the generalizability of the McNevin et al. (2003) findings. In the Porter et al. experiment, low-skilled subjects were provided instructions that prompted them to focus their attention externally at systematically increasing distances prior to performing a standing long jump. Consistent with the result reported by McNevin et al. (2003), the results of the Porter et al. experiment demonstrated that participants’ jumping performance was enhanced as they directed their attention externally at increasingly greater distances. These findings suggest that the benefits of an external FOA are magnified as attention is directed at greater distances from the body.

The constrained action hypothesis is frequently used to explain the benefits of adopting an external rather than an internal focus during motor skill performance (Wulf et al., 2001a). This viewpoint suggests when a mover directs their attention externally the motor control system self-organizes more effectively, facilitating movements to be controlled automatically in a non-conscious manner. Whereas, when an internal FOA is utilized motor behaviors are consciously controlled; this causes the motor control system to become constrained, consequently depressing motor performance. Several studies have been conducted providing support for the predictions of the constrained action hypothesis (Wulf et al., 2001a, 2010; Marchant et al., 2009; Lohse et al., 2010; Makaruk et al., 2012).

It is presently not well understood why instructing an individual to direct their attention at increasingly greater distances (e.g., McNevin et al., 2003; Porter et al., 2012) enhances motor performance. One possible reason is movers plan the execution of a task with the desired outcome in mind. Hence, directing attention toward a distally located object may facilitate the more optimal production of preplanned movements; as would be the case when performing a discrete motor skill (e.g., standing long jump). Also, monitoring movements in terms of their distal effects likely diminishes the on-line monitoring of continuous tasks (e.g., balancing on a stabilometer), resulting in a less constrained motor control system. Additionally, it is also possible that directing attention toward more distant effects aligns the mover’s visual attention with their cognitive attention. One goal of the present study was to better understand how manipulating the distance of attention with and without vision impacts motor behavior. Doing this will not only assist in establishing the underlying mechanisms of the “distance” effect, but will also help clarify the role vision plays in how conscious attention is directed. Presently, the interaction between vision and the distance of FOA has not been investigated. Understanding this interaction will bring valuable understanding to the underlying cause of the FOA effects reported in previous research.

Although there are numerous studies that consistently demonstrate the benefits of adopting an external FOA, little is actually understood about the underlying mechanisms that govern the effect. One avenue that has not been adequately investigated is how vision interacts with the movers’ FOA. Specifically, it is not understood if a person’s ability to see a target is a mitigating factor to properly focusing attention internally or externally. Moreover, it is not understood if adopting an external FOA is dependent on a person’s ability to see the “external” result of the movement. Understanding the role vision plays when focusing attention is critical to advance the mechanistic understanding of the influence a mover’s attentional focus has on performance. Some researchers have tried to control vision across conditions by instructing participants to close their eyes during the performance of all trials (McNevin and Wulf, 2002), fixate on a specific target during task execution (Porter and Anton, 2011), or to look straight ahead while performing the prescribed task (Wulf et al., 2001a). To date, no studies have occluded a portion of the visual field while simultaneously manipulating a participant’s FOA. Inhibiting a participant’s ability to see critical aspects of a task on some trials, while allowing full vision on other trials across different FOA conditions (e.g., internal, external-near, external-far) would provide valuable insight from both a theoretical and practical perspective about the contributory role vision plays in the FOA effect.

The purpose of this study was twofold. First, we sought to investigate if vision or the lack thereof influenced motor performance when participants were instructed to adopt either an internal FOA, an external FOA near the body, or an external FOA located at a greater distance from the body. Second we were interested if increasing the distance of an external FOA from the body was mitigated by the visual system. Understanding the role of the visual system (i.e., visual attention) and how it contributes to (or possibly inhibits) FOA (i.e., cognitive attention) is paramount to more fully understand the underlying mechanisms that are causing the differences in motor behavior so frequently reported in the scientific literature.

We investigated these questions with a manual tracking task that is modeled after previous work on visuomotor coordination, and in particular, which incorporates three key features. First, the participant tracks a target that moves along an unpredictable, non-linear path (e.g., Becker and Fuchs, 1985; Engel et al., 2000). Second, before each trial, participants are provided with visuo-spatial cues that indicate the upcoming motion path (e.g., Elliott and Madalena, 1987; Westwood et al., 2003; Binstead et al., 2006). And third, on some trials the target undergoes transient occlusion (e.g., Olson et al., 2003; Bennett and Barnes, 2006; Mrotek and Soechting, 2007). Together, these task features allow us to systematically examine the role of spatial working memory during visuomotor coordination, by requiring participants to not only encode the pre-trial visual cues, but also use these cues as they track both visible and occluded targets.

In particular, participants in the current study used a computer mouse to track visible and occluded targets. In addition, the distance effect was examined by comparing tracking performance across three FOA groups: an internal group, which was instructed to move “your hand as smoothly and evenly as possible,” and external-near and external-far groups, who received comparable instructions but were told to focus on moving “the mouse” or “the cursor,” respectively. Two measures of tracking performance were analyzed: mean tracking error (i.e., the distance from the onscreen cursor to the target) and mean power frequency (MPF, derived from participants’ tracking error time series). While mean tracking error provides a direct index of effectiveness on the task, MPF is a more indirect measure that characterizes the distribution of frequencies underlying participants’ movements. In particular, the constrained action hypothesis posits that increases in MPF across task conditions indicate a greater reliance on subconscious or automatic control processes (e.g., Wulf et al., 2001b; McNevin et al., 2003).

Given the ubiquitous finding that an external FOA enhances movement accuracy, we first predicted that tracking errors would be lowest in the external-far condition, while the internal condition would have the largest tracking errors. Second, given that the benefit of an external FOA is modulated by the distance from the individual to the movement effect, we also predicted that tracking error in the external-near condition would be between the other two FOA conditions. Third, we predicted the reverse ordering for the analysis of MPF: highest MPF in the external-far FOA condition, followed by the external-near and then internal conditions. Finally, we also predicted target visibility would not mediate the influence of FOA on tracking performance, that is, the same pattern of results would be observed across visible and occluded trials.

## Materials and Methods

### Participants

All participants were recruited from an undergraduate introductory psychology course and received course credit for the study. A total of 114 students participated (75 females; 39 males). As we highlight below, two performance exclusion criteria were applied to the sample, resulting in a final total of 98 participants (66 females; 32 males). All participants completed an informed-consent form prior to the study, and after completing the study, participants received a debriefing that explained the purpose and goals of the study.

### Stimuli and apparatus

**Figure 1:**
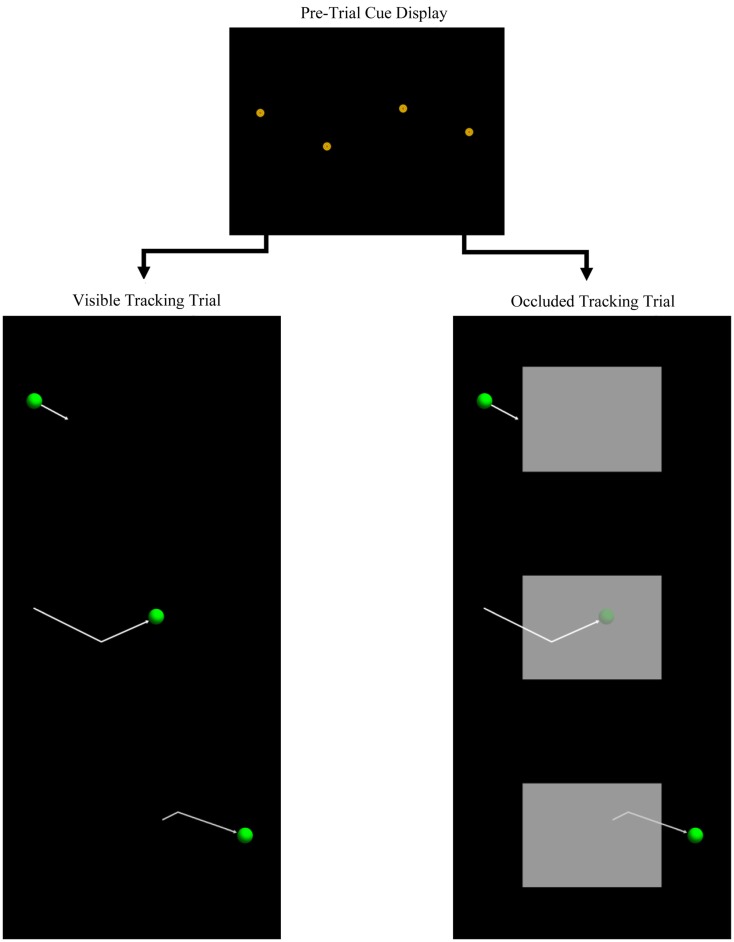
**Schematic diagram of the manual tracking task**. The top figure illustrates a pre-trial cue display, while the left, and right panels illustrate two tracking trials that would follow the pre-trial cue display. The arrows are for illustration purposes only, as well as the semi-transparent target in the occluded trial (right panel).

Figure 1 illustrates the stimuli used for the tracking task: (1) a circular, gold and brown bull’s-eye shape, (2) a green sphere, and (3) a gray rectangle. Stimulus presentation was controlled with the E-Prime (Psychology Software Tools) software suite, and displayed on a 21′′ CRT display with the resolution set at 1024 × 768 pixels. In screen coordinates, the size of the three stimuli were 29 and 58 pixels for the diameter of the bull’s-eye and sphere, respectively, while the rectangle was 512 × 384 pixels. Given the viewing distance of 24 in (61 cm), the visual angles of the stimuli were therefore 1.1° and 2.2° for the bull’s-eye and sphere, respectively, and 19.7° × 14.8° for the rectangle. Participants were tested on Dell Vostro 200 workstations running Windows XP, and used the mouse and keyboard to respond.

### Procedure

At the start of the testing session, participants were instructed to use the mouse to control the movement of the onscreen cursor. The session was then divided into two tasks. First, to help participants learn to attend to and encode the locations of the pre-trial spatial cues, a short spatial-memory test was presented, in which four of the bull’s-eye shapes were briefly presented onscreen in a lateral “zig-zag” pattern (see Figure 1) and then removed. Participants then indicated the locations of the four shapes. A total of 24 spatial-memory test trials were presented, with the shapes randomly positioned on each trial.

Next, participants were presented with the manual tracking task. In order to help familiarize participants with this task, a practice trial was presented first. During the practice tracking trial, the target (green sphere) moved slowly (i.e., 333 pixels/s) along a horizontal path, while fully visible. Participants were instructed to “keep the cursor as close to the target as possible while it moves.” Feedback was provided during the practice trial by varying the color of the target. In particular, the target changed to red when contacted by the cursor, and then back to green when the cursor was off the target. Note that the target only changed color during the practice trial, and otherwise remained green during all subsequent test trials.

Participants then completed 60 tracking test trials, divided into five blocks of 12 trials. Each test trial was composed of a pre-trial cue phase, followed by a tracking phase. Figure 1 illustrates two examples of the cue and tracking phases (note that motion arrows are for illustration purposes and were not presented during testing). During the pre-trial cue phase, the four spatial cues were presented for 2 s. Cues were randomly positioned on each trial, subject to three constraints: (1) total path distance from the first to the fourth cue was equal to the width of the display (i.e., 1024 pixels), (2) the distance between adjacent cues was fixed at 1/3 of the display width (i.e., 341 pixels), and (3) the change in path orientation from one cue to the next varied randomly between 15° and 45°.

The pre-trial cues were displayed for 2 s and then removed. Next, the tracking target appeared, centered on the previous location of either the left- or right-most cue. The target then moved at a constant speed along the path specified by the pre-trial cues. Initial position of the target (left versus right side of the display) and direction of motion was counterbalanced across trials. Duration of target motion was 4 s, resulting in a constant speed of 256 pixels/s (9.8°/s). Target position was updated at the rate of 24 fps.

During half of the test trials, a large occluder (i.e., the gray rectangle) was also displayed, centered on the screen. On occluded trials, the target passed behind the occluder and reappeared on the other side (see Figure 1; note that the occluded target is illustrated here as semi-transparent to indicate its location behind the occluder). Occluded and visible trials varied randomly and were counterbalanced within each of the five blocks.

Participants were randomly assigned to one of three FOA instruction conditions: internal, external-near, or external-far. In the internal condition, participants were instructed to “please pay attention to how *your hand* moves. In particular, try to keep *your hand* moving smoothly and evenly at the same speed” (italics added here for emphasis). Participants in the other two conditions were given equivalent instructions, with a substitution of two words for “your hand”: that is, “the mouse” or “the cursor” in the external-near and external-far conditions, respectively. FOA instructions were presented onscreen at the start of each block of test trials.

### Data collection

Cursor position was sampled 24 times per second (24 Hz) while participants tracked the target. Two dependent measures were derived from cursor position: mean tracking error and MPF. Tracking error was defined as the Euclidean distance from the center of the cursor to the center of the target. For each trial, mean tracking error was computed as the average tracking error over the path of the target. Note that for the occluded trials, mean tracking error was restricted to the occluded portion of the target path (i.e., the visible portion was not included in the analysis). For consistency, therefore, analysis of the visible trials was also restricted to the corresponding portion of the target path (i.e., roughly the middle 50%).

The second dependent measure was MPF, which was computed by mapping the time series sequence of tracking errors into the frequency domain via the fast Fourier transform (i.e., FFT). Next, the output of the FFT (i.e., amplitude spectrum) was converted to power, and finally, the mean frequency of the resulting power distribution was computed.

In order to address the presence of sporadic outliers in the data set (as well as occasional participants who did not consistently comply with task instructions), two exclusion criteria were applied to the dataset prior to analysis. First, recall that each block included six occluded trials and six visible trials. In order to focus on participants’ optimal performance, the two occluded trials within each block with the highest mean tracking errors were dropped for each participant. The same exclusion rule was also applied to the visible trials, resulting in four occluded trials and four visible trials per participant per block (i.e., 40 samples per participant). Next, non-compliant subjects were excluded by inspecting the resulting dataset: non-compliance was defined as failure to track the target (i.e., not moving the mouse for at least 50% of the trial), on 25% or more of the trials. Using this criterion, four participants were excluded from the external-far and internal conditions, and eight participants from the external-near condition, resulting in 34, 34, and 30 participants, respectively, retained in each condition^1^.

We evaluated our hypotheses by performing two four-way ANOVAs. The first analysis was a 5 (Block) × 4 (Trial) × 2 (Occlusion) × 3 (FOA) mixed-model ANOVA, with the first three factors as within-subjects and the last as between-subjects, and mean tracking error as the dependent measure. The second analysis followed the same design, with MPF as the dependent measure. We also conducted a supplementary analysis, which evaluated the correlation between mean tracking errors and MPF.

## Results

### Analysis of mean tracking errors

**Figure 2:**
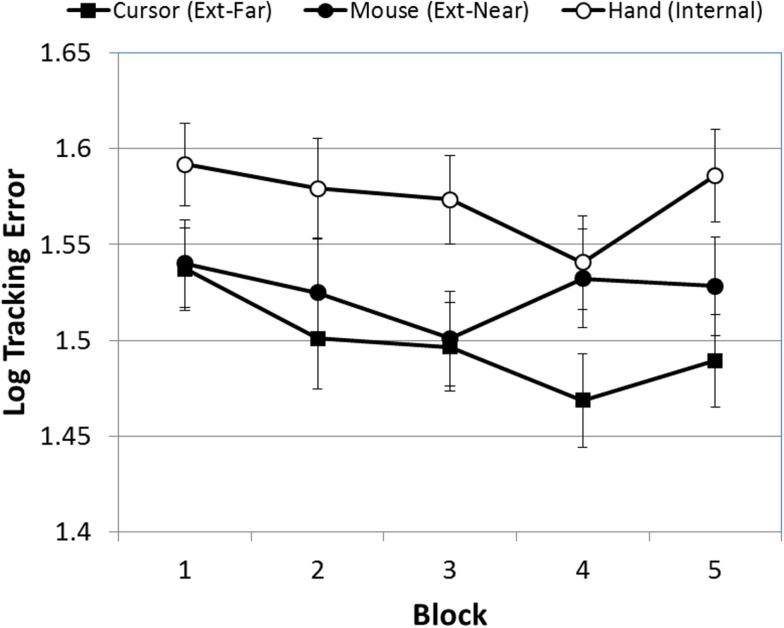
**Mean tracking error (log-transformed) across blocks, as a function of FOA condition (error bars are ±1 SE)**.

Preliminary analyses revealed significant deviations from normality in the two dependent measures. As a result, both datasets were log-transformed prior to analysis. The first ANOVA evaluated the effect of block, occlusion, FOA, and trial on participants’ mean tracking errors. Figure 2 presents mean tracking errors over blocks, as a function of FOA. As Figure 2 indicates, there was a significant effect of block [*F*(4, 380) = 4.82, *p* < 0.001]. A *post hoc* pairwise comparison (Least Significant Difference) of the five test blocks confirmed that tracking errors during the first block of trials were significantly higher than the subsequent three blocks, and that the fourth block was significantly lower than the fifth block. Second, improvement on the task did not vary across FOA condition: the Block × FOA interaction was not significant [*F*(8, 380) = 1.52, *p* > 0.05].

**Figure 3:**
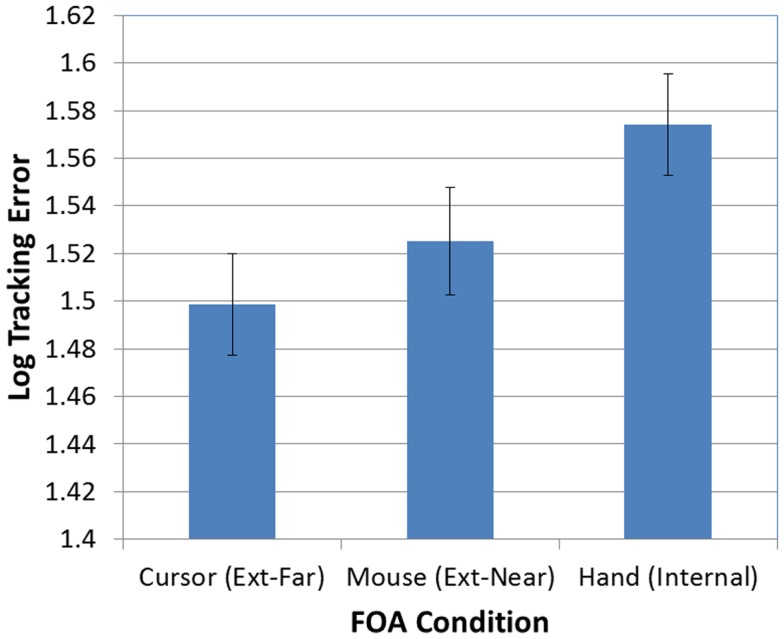
**Mean tracking error (log-transformed) as a function of FOA condition (error bars are ±1 SE)**.

**Figure 4:**
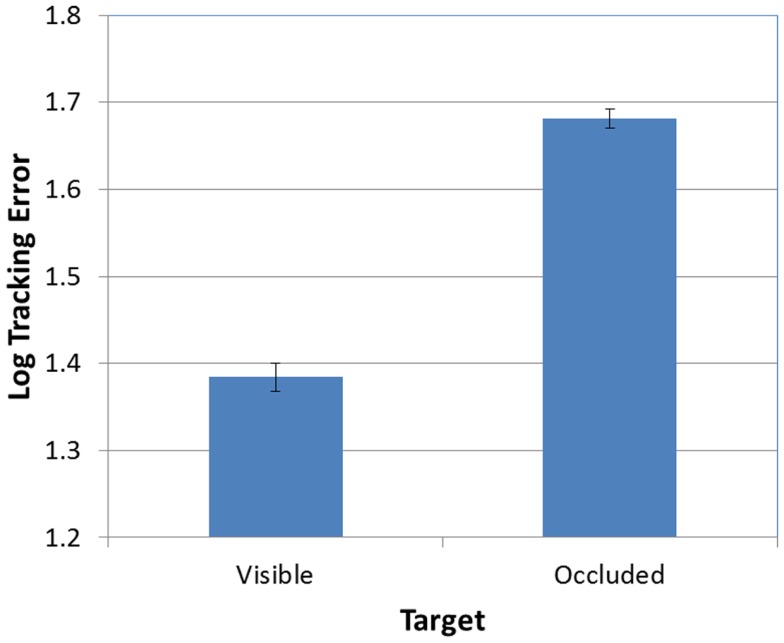
**Mean tracking error (log-transformed) as a function of target visibility (error bars are ±1 SE)**.

Next, as predicted, the effect of FOA was significant [*F*(2, 95) = 3.26, *p* < 0.05]. Figure 3 presents participants’ mean tracking errors as a function of the FOA instructions. *Post hoc* comparisons revealed that participants in the external-far condition produced significantly lower tracking errors than participants in the internal condition. Meanwhile, tracking errors in the external-near condition fell midway between, and did not differ significantly from, the external-far and internal conditions. In addition, the effect of occlusion was significant [*F*(1, 95) = 714.66, *p* < 0.001]. As Figure 4 illustrates, participants’ tracking errors were significantly lower when the target was visible.

**Figure 5:**
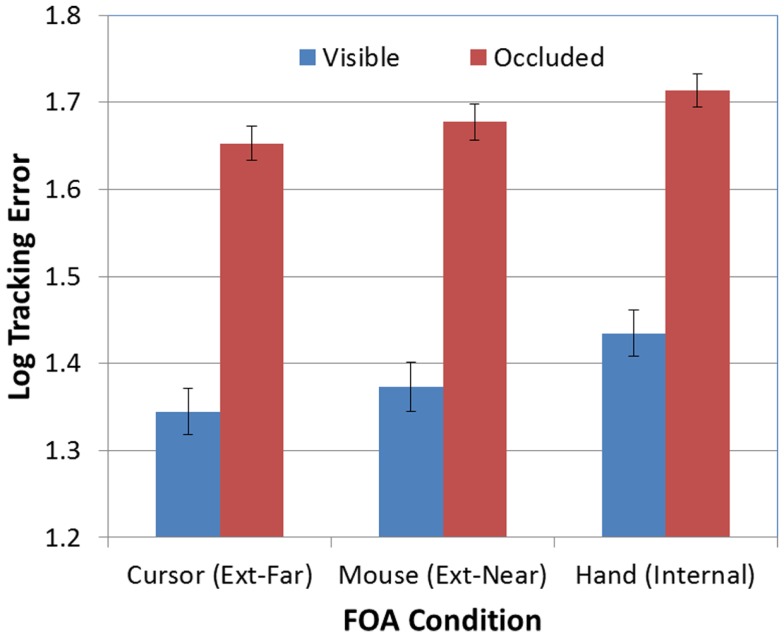
**Mean tracking error (log-transformed) as a function of FOA condition and target visibility (error bars are ±1 SE)**.

Finally, there was not a significant FOA × Occlusion interaction [*F*(2, 95) = 0.70, *p* > 0.05]. Thus, as predicted, the effect of FOA on tracking errors was not moderated by the visibility of the target. Whether tracking was visually guided or memory-guided, participants in the external-far condition produced lower tracking errors than participants in the other two FOA conditions, while participants in the internal condition tended to produce the highest tracking errors (see Figure 5).

Analysis of the remaining interactions revealed that only the Trial × FOA interaction was significant [*F*(2, 285) = 2.41, *p* < 0.03]. Simple-effects tests of the effect of trial on mean tracking errors for each of the FOA conditions indicated that participants in the internal condition tended to improve across trials within each block, while in both of the external conditions there was no clear pattern of performance across trials.

### Analysis of MPF

**Figure 6:**
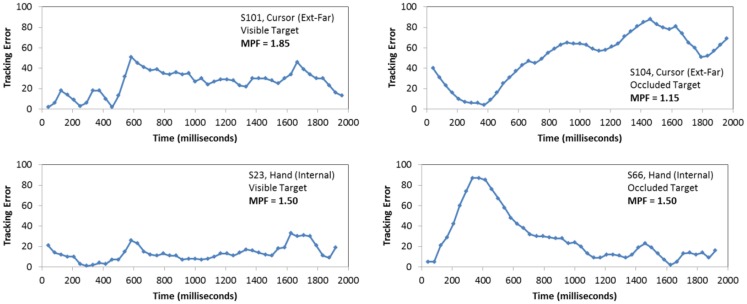
**Examples of performance on the manual tracking task**. Each plot presents the tracking error (i.e., distance from the cursor to the center of the target) produced during the trial, as a function of time. The upper two samples illustrate high and low MPF values during visible and occluded trials, respectively, while the bottom two illustrate moderate levels of MPF during corresponding trials.

The second ANOVA evaluated the effect of block, occlusion, FOA, and trial on participants’ MPF. Recall that higher MPF values across conditions reflect a relative increase in higher-frequency movements during the tracking task. In particular, such increases are taken as evidence of greater reliance on intrinsic movement dynamics, or in other words, recruitment of automatic control processes for movement. To help illustrate how MPF values varied across participants and trial types, Figure 6 presents four examples of tracking error time series. The upper two plots provide examples of high and low MPF values during visible and occluded trials, respectively, while the bottom two plots illustrate moderate levels of MPF during corresponding trials.

**Figure 7:**
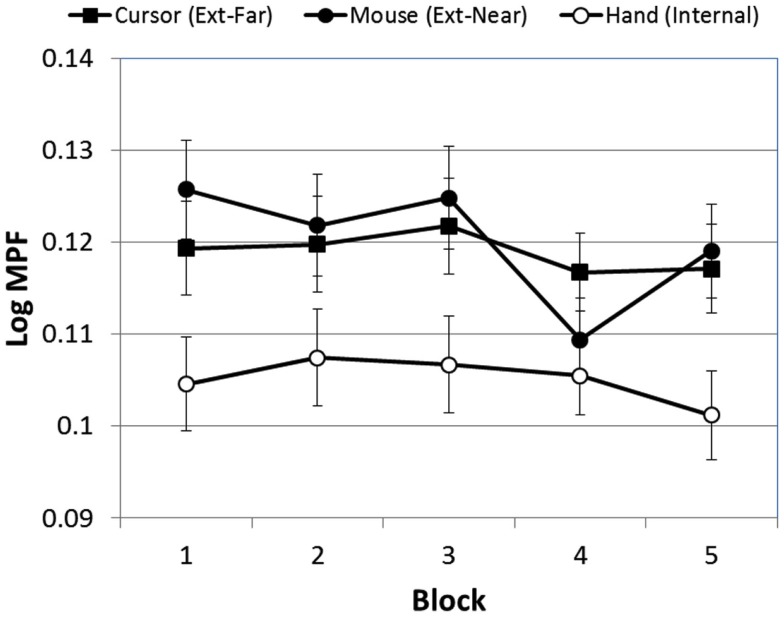
**Mean MPF (log-transformed) across blocks, as a function of FOA condition (error bars are ±1 SE)**.

First, MPF varied significantly over blocks [*F*(4, 380) = 2.58, *p* < 0.03]. The linear trend for this effect, however, was only marginally significant [*F*(1, 95) = 3.83, *p* < 0.06]. Although the Block × FOA interaction was not significant [*F*(8, 380) = 1.09, *p* > 0.05], Figure 7 suggests that the external-near condition was largely responsible for this effect. We therefore conducted an exploratory simple-effects test of the effect of block on tracking errors within each FOA condition, and found a significant effect of block only in the external-near condition [*F*(4, 116) = 3.39, *p* < 0.02]. A subsequent analysis of linear and polynomial trends in this condition revealed only a significant forth-order effect [*F*(8, 380) = 1.09, *p* < 0.05], suggesting that MPF did not increase or decrease monotonically in any of the FOA conditions.

**Figure 8:**
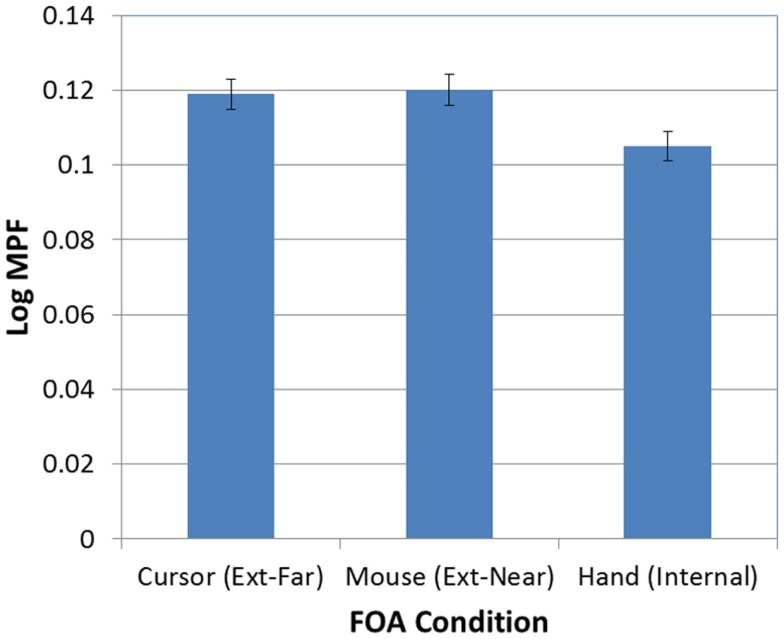
**Mean MPF (log-transformed) as a function of FOA condition (error bars are ±1 SE)**.

Next, as expected, there was a significant effect of FOA on participants’ MPF [*F*(2, 95) = 4.30, *p* < 0.02]. As Figure 8 illustrates, MPF was significantly higher in the two external FOA conditions than in the internal FOA condition. In addition, MPF did not differ in the external-near and external-far conditions (i.e., the effect of external FOA distance on MPF was not significant).

**Figure 9:**
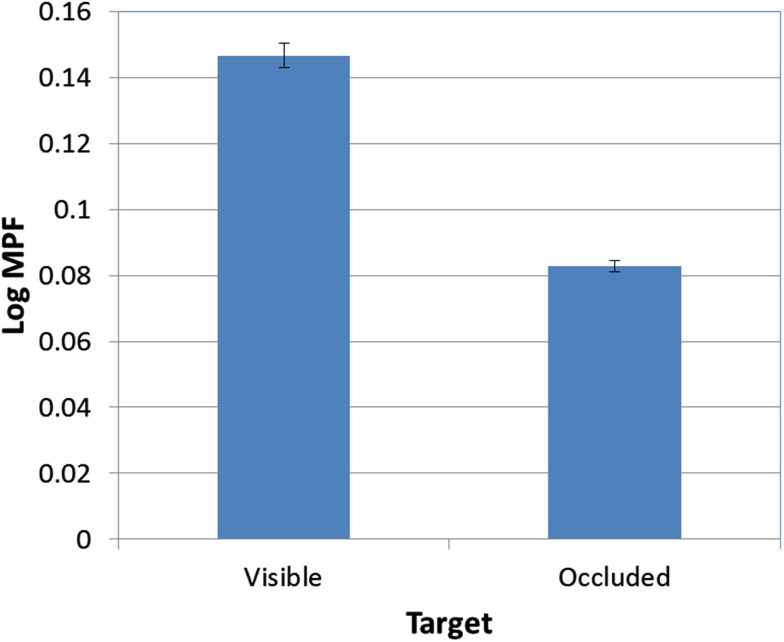
**Mean MPF (log-transformed) as a function of target visibility (error bars are ±1 SE)**.

**Figure 10:**
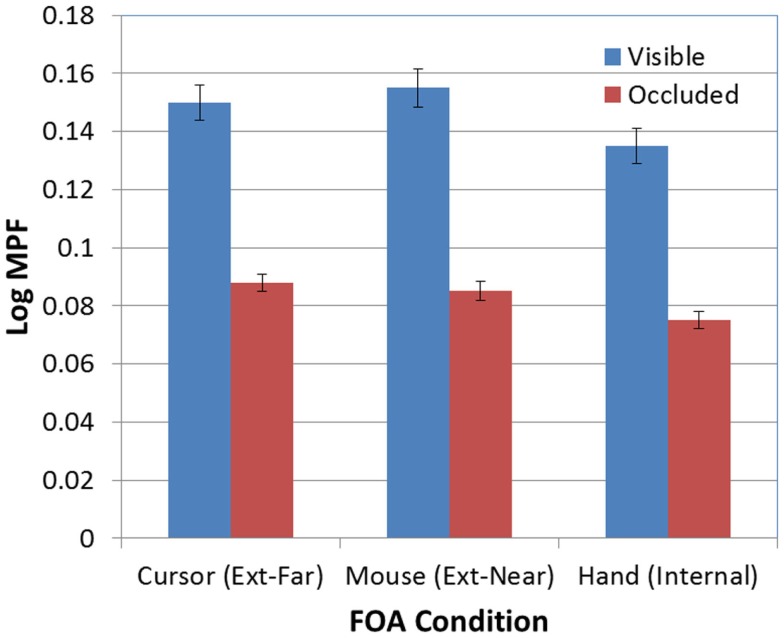
**Mean MPF (log-transformed) as a function of FOA condition and target visibility (error bars are ±1 SE)**.

There was also a significant effect of occlusion on MPF [*F*(1, 95) = 389.60, *p* < 0.001; see Figure 9]. In addition, the FOA × Occlusion interaction [*F*(2, 95) = 0.83, *p* > 0.05; see Figure 10] was not significant, indicating that the effect of occlusion on MPF did not vary as a function of FOA. Therefore, across all three FOA conditions, participants’ MPF was significantly higher when tracking visual targets than when tracking occluded targets. However, the main effect of occlusion on MPF was qualified by a 2-way Block × Occlusion interaction [*F*(4, 380) = 3.24, *p* < 0.02]. This interaction reflected the tendency for MPF to decline across blocks during visible trials, while remaining relatively constant during occluded trials. In addition, there was also a significant 3-way Occlusion × Trial × FOA interaction [*F*(6, 285) = 2.18, *p* < 0.05]. In particular, MPF trended downward across occluded trials in the external-near condition, while it remained comparatively more level across both occluded and visible trials in the external-far and internal conditions. No other main effects or interactions were significant.

Finally, we conducted a supplementary analysis that examined the association between mean tracking errors and MPF by computing the correlation between the two measures. In particular, this analysis allowed us to assess and validate a key assumption of the constrained action hypothesis, that is, that higher levels of MPF reflect automatic corrective movements. If this assumption is correct, MPF and tracking errors should therefore be negatively correlated. Indeed, as Table 1 indicates, there were strong negative associations between tracking errors and MPF for both the visible and occluded trials [i.e., *r*(96) = −0.789 and −0.472, respectively, both *p*s < 0.001]. In addition, the strength of the association was greater for the visible trials than for the occluded trials [Fisher’s *z*-test; *z*(190) = 2.18, *p* < 0.01]. However, pairwise comparisons between the three FOA conditions revealed no statistically significant differences, during either the visible or occluded trials (all *z*s < 0.65, *p*s > 0.10).

**Table 1:** **Mean tracking error – MPF correlations**.

FOA condition	Target condition	
	Visible	Occluded
Cursor (external-far)	−0.671***	−0.382*
Mouse (external-near)	−0.826***	−0.484**
Hand (internal)	−0.833***	−0.480**
All conditions	−0.789***	−0.472***

***p < 0.001, **p < 0.01, *p < 0.05.

## Discussion

Our primary goal in the current study was to examine the role of visual feedback during manual tracking, and in particular, to determine whether visibility of the moving target would mediate the influence of an internal versus external FOA on tracking performance. We hypothesized that an external FOA would result in better tracking performance than an internal FOA, and that focusing on external effects farther from the body would result in better performance than focusing on external effects near the body. More importantly, we assessed the role of vision as a mechanism that underlies or supports the influence of FOA, by comparing performance across visible and occluded trials. Critically, we hypothesized that the same pattern of results would be observed during tracking of both visible and occluded targets. Taken together, the results provided broad support for our predictions.

First, as expected, participants who were instructed to focus on external effects farthest from their bodies (i.e., the onscreen cursor) had significantly lower tracking errors than those who had an internal focus (i.e., their hand). Participants who were instructed to focus on a location near but external to their body (i.e., the mouse) performed at a level intermediate between the other two conditions. Analysis of MPF confirmed the influence of external versus internal FOA, although the effect on participants’ MPF in the two external conditions was not mediated by the distance of focus. Thus, participants who focused on producing external effects generated higher levels of MPF than those who focused on internal effects, regardless of whether the external focus was near to or far from the body.

Second, we also found that tracking performance varied as a function of target visibility. Perhaps not surprisingly, participants tracked visible targets more successfully than occluded targets. A related and more important finding, however, is that MPF also varied with target visibility: specifically, MPF was significantly higher when the target was visible. This finding strengthens the proposal that increases in MPF are linked to the production of high-frequency corrective movements (e.g., Wulf et al., 2001a; McNevin et al., 2003), and in particular, it suggests that these movements are triggered more often when both the cursor and the target are visible, than when only the cursor is visible. Additional support for this proposal was also provided by not only the finding that mean tracking errors and MPF were negatively correlated, but also that the associative pattern between tracking errors and MPF was significantly stronger when the target was visible than when it was occluded. Given these findings, we should therefore predict that additional kinematic analyses will reveal more frequent corrective movements when the target is visible.

A third and related issue concerns how performance changed over time. Indeed, tracking errors declined over blocks, reflecting an improvement in tracking performance with practice. Based on the finding that corrective movements decrease in frequency with practice on movement tasks (e.g., Sherwood and Canabal, 1988), a relatively straightforward prediction is that MPF – which is a putative index for corrective movements – should also decrease with practice. However, we did not observe a clear pattern of changes in MPF (i.e., either increasing or decreasing) over blocks, or over trials within each block. Thus, the present findings do not provide a consistent picture of whether and/or how MPF varies with practice. The lack of a clear result is somewhat mirrored by the mixed findings that have emerged from studies which have examined other variants of MPF, for example physiological measures such as EMG (e.g., Vance et al., 2004; Lohse et al., 2010). This issue remains an open question.

Fourth, and most relevant to our primary goal, we confirmed the prediction that varying the visibility of the target would not mediate the influence of FOA on tracking performance. Indeed, regardless of whether participants tracked visible or occluded targets, the instructions to focus on either the internal or external effects of movement led to the same pattern of results. Given that target visibility had an acute impact on tracking errors and MPF, the finding suggests a potential dissociation between the role of vision (and visual feedback) on task performance, and its role as a mechanism that underlies attentional focus during motor performance. One implication that follows from this work is that visual focus (i.e., on-line visual focusing) can be dissociated from cognitive focus, and that the latter may be tied to mechanisms that support cognitive functions which do not require ongoing and direct visual input, such as mental imagery, prospective action, and episodic memory.

There are also a number of ways that the current study can be extended and elaborated to help identify the mechanisms that underlie the FOA effect. First, it is important to acknowledge that participants performed best in the external-far condition, in which they were instructed to focus on the movement of the cursor. As mentioned in the introduction, the elevated performance in this condition may have been the result of more effective on-line process as the result of a less constrained motor system. However it is also possible that their skill in this condition may have been exploited, at least in part, because of everyday experience with using a computer mouse while attending to the position and movement of the cursor. One way to reduce this bias may be to provide participants with another means to control cursor position, such as moving their arm and hand while holding a wireless pointing device (e.g., Wii remote) Clearly additional research is needed to fully explain the distal effects reported in this and previous studies. Second, as we noted above, recording and analysis of kinematic measures such as movement units and corrective movements will not only verify the assumption that increases in high-frequency movements are in fact corrective, but they may also help to demonstrate a causal relation between changes in MPF and corrective movements. Such analyses may also help to address the question of whether MPF reliably changes with practice. Third, a straightforward extension of our paradigm involves introducing a concurrent secondary task, in order to demonstrate that participants have greater free cognitive resources when instructed to utilize an external FOA (i.e., they are less susceptible to interference from the secondary task). Finally, the sources and amount of visual feedback available while tracking can also be systematically varied, for example, by creating an aperture in the occluding screen or intermittently “flashing” the target while occluded (e.g., Elliott et al., 1995).

## Conflict of Interest Statement

The authors declare that the research was conducted in the absence of any commercial or financial relationships that could be construed as a potential conflict of interest.

## Footnotes

^1^We examined the question of whether differential attrition in the three FOA conditions biased the results by conducting a supplementary analysis, in which the number of participants per condition was equated by dropping the four lowest-performing participants in the external-far and internal conditions. The qualitative pattern of findings from the subsequent analysis mirrored the findings from our original analysis.
